# Usefulness of 3D transperineal ultrasound in severe stenosis of the anal canal: preliminary experience in four cases

**DOI:** 10.1007/s10151-013-1078-8

**Published:** 2013-10-01

**Authors:** M. Kołodziejczak, G. A. Santoro, R. Z. Słapa, T. Szopiński, I. Sudoł-Szopińska

**Affiliations:** 1 Department of Proctology, Hospital at Solec, 00-382 Warsaw, Poland; 2 Pelvic Floor Unit, I Department of Surgery, Regional Hospital, Piazzale Ospedale 1, 31100 Treviso, Italy; 3 Department of Diagnostic Imaging, Medical University of Warsaw, Kondratowicza St. 8, 03-242 Warsaw, Poland; 4 Clinic of Urology, Collegium Medicum of the Jagiellonian University, Kraków, Poland; 5 Department of Radiology, Institute of Rheumatology, Warsaw, Poland

**Keywords:** Anal stenosis, Three-dimensional ultrasound, Transperineal ultrasound

## Abstract

**Background:**

Organic or functional anal canal stenoses are uncommon conditions that occur in the majority of cases as a consequence of anal diseases. A proper assessment is fundamental for decision making; however, proctological examination and endoanal ultrasound are often unfeasible or very difficult to perform even under local or general anesthesia. We therefore began to use 3D transperineal ultrasound to assess patients. The aim of this study was to compare the results of evacuation proctography and 3D transperineal ultrasound in patients with severe anal canal stenosis.

**Methods:**

Four consecutive patients with high-grade anal canal stenosis were evaluated using both proctography and 3D transperineal ultrasound with a micro-convex transducer between March and June 2011.

**Results:**

In all cases, 3D transperineal ultrasound provided detailed information on the length and level of stenosis and on the integrity of the anal sphincters.

**Conclusions:**

Our preliminary experience suggests that 3D transperineal ultrasound makes it possible to plan optimal surgical treatment.

## Introduction

Anal canal stenosis is an uncommon condition. Only rarely is it primary, due to congenital malformations. The majority of cases develop as a consequence of anal diseases leading to organic or functional stenosis [1]. Organic stenosis (i.e., stricture) typically results from scarring following crypt-related inflammatory diseases (abscess, fistula), inflammatory bowel disease, overly extensive hemorrhoidectomy, radiation therapy to the anorectal area or pelvis, anal canal trauma, or a stenotic tumor [1]. Functional stenosis is caused by internal anal sphincter hypertonus related to anal fissure or inflammation of the anal crypt leading to sphincter hypertrophy [2–7].

The most common three-grade classification of anal stenosis takes into account the results of rectal examination and evaluation with the Hill–Fergusson retractor [8]. Patients with first- and second-degree stenoses may be treated conservatively, while third-degree stenosis requires surgical correction.

Preoperative assessment is fundamental for surgical planning; however, the digital rectal examination, under local or general anesthesia, is often unfeasible or very difficult. The most relevant information that the surgeon needs are length and level of the stenosis, i.e., whether it is limited to the anal canal or includes the rectum, and the status of the anal sphincters. Endoanal ultrasound (US) is the gold standard investigation for the evaluation of the anal canal [9–21]; however, it cannot be used in patients with severe stenosis. Transperineal US has been used as an alternative modality for the evaluation of the anal canal, with good agreement compared to endoanal US [22–28]. Proctography can be also useful for the assessment of the length of a stenosis in the anal canal.

We report our preliminary experience on the usefulness of three-dimensional (3D) transperineal US compared to proctography in surgical decision making in 4 cases of severe anal canal stenosis.

## Materials and methods

Four consecutive patients with high-grade anal canal stenosis (one with a second-degree and three with a third-degree stenosis) were evaluated between March and June 2011. The three-grade classification of anal stenosis used in the study took into account the results of rectal examination and evaluation with the Hill–Fergusson retractor [8], where first-degree stenosis is a mild stenosis when digital rectal examination is feasible with the use of a lubricant or middle-size Hill–Fergusson retractor; second-degree stenosis—inability to introduce the index finger nor a mild Hill–Fergusson retractor into anal canal; third-degree stenosis—inability to introduce the 5th finger nor small Hill–Fergusson retractor.

The initial clinical patient evaluation and treatment were conducted at the Department of Proctology, Hospital at Solec, whereas ultrasound examinations were performed in the Department of Diagnostic Imaging, Medical University of Warsaw.

The protocol was approved by the Medical University’s review board, and all participants gave written informed consent.

### Imaging techniques

In all cases, proctography with the administration of uropolinum through a thin catheter was performed prior to ultrasound examination.

**Fig. 1:**
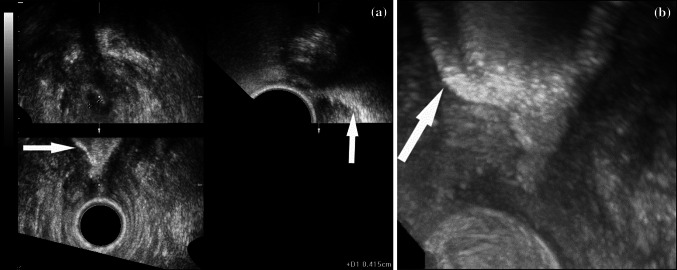
**a** Multiplanar reconstruction: on axial plane (*upper left*), irregular outlines of the anal canal are visible (between calipers); on sagittal (*upper right*) and coronal (*lower*) planes, short (1 cm long) stenosis of the anal canal with distended rectal ampulla (*arrow*) is visible; **b** maximum intensity projection: distended rectal ampulla (*arrow*) and proximal part of the anal canal above stenosis

**Fig. 2:**
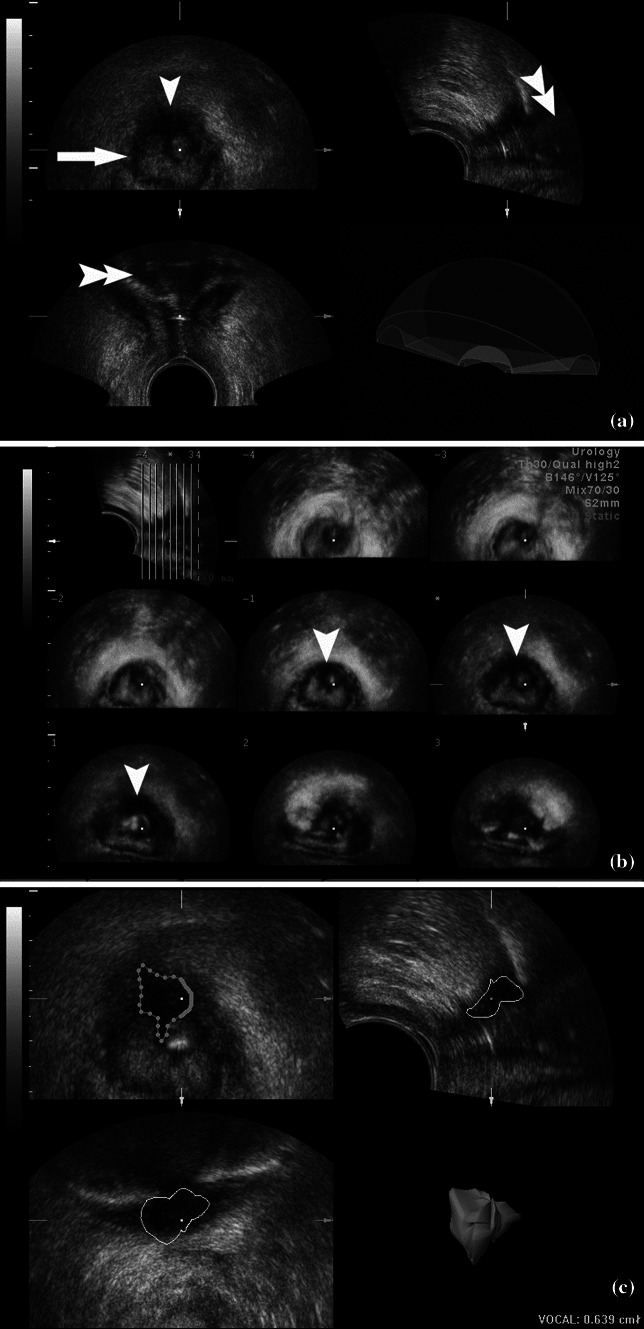
**a** Multiplanar reconstruction: on axial view (*upper left*), a thin circular (*arrow*) scar in the external anal sphincter and a bulky scar (*arrowhead*) that involves both sphincters and is adherent to the anoderm are seen. Short (1 cm long) obstruction with distended rectal ampulla (*double arrowheads*) is visible on sagittal (*upper right*) and coronal (*lower*) planes. **b** Tomographic ultrasound imaging with static volume contrast imaging. Bulky scar (*arrowheads*) on consecutive axial slices seen in the *upper* part of anal canal (see the *pilot sagittal image*—*upper left*). **c** Evaluation of volume of the bulky scar and its 3D presentation (*lower right*)

**Fig. 3:**
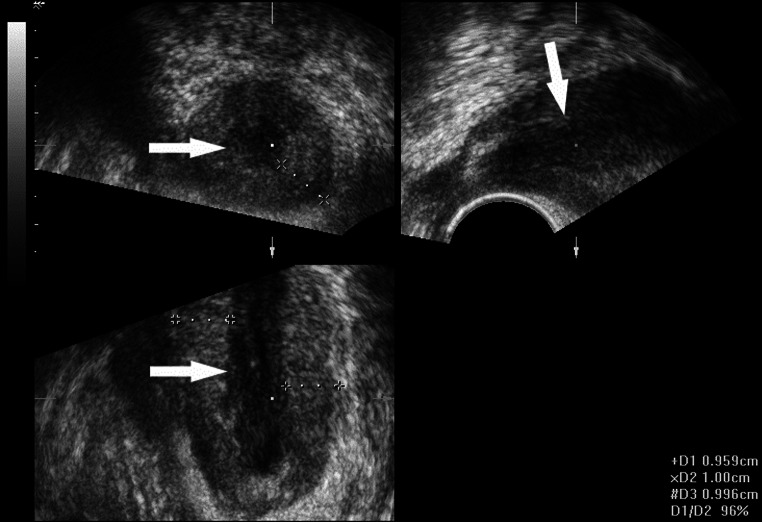
Multiplanar reconstruction: axial, sagittal, and coronal views of anal canal show hypertrophy of internal anal sphincter (10 mm thickness; calipers) and hypoechoic mucosa (*arrows*) devoid of normal folded pattern
- Tomographic US imaging, like CT or MRI scans, enables visualization of the whole anal canal or just the lesion in consecutive planes (e.g., axial) to optimally present the whole pathology in one display (Fig. 2b);
- Volume render mode enables the assessment of the content of 3D presentation of the anal canal and surrounding tissues more precisely [11], using different post-processing techniques:
  - Minimum intensity projection, which employs an algorithm displaying an interactive (e.g., with 360° rotation around the longitudinal axis of the anal canal) 3D image of dark (hypoechoic or anechoic) structures, e.g., hypoechoic internal anal sphincter or hypoechoic scar.
  - Maximum intensity projection, which employs an algorithm displaying an interactive (e.g., with 360° rotation around the longitudinal axis of the anal canal) 3D image of bright (hyperechoic) structures, e.g., hyperechoic contents of a distended rectal ampulla in a patient with anal canal stenosis (Fig. 1b). Such images may resemble radiologic proctography with uropolinum.
  - Static volume contrast imaging, which is a thin slice volume rendering technique with combination of various algorithms, including surface algorithm (Fig. 2b). It renders slices as thick as 2–10 mm and provides higher contrast and less noise than a conventional display.

Transperineal US was performed using a Voluson 730 scanner (General Electric Medical Systems, Kretz Ultrasound, Zipf, Austria) with a multifrequency (3.3–10 MHz) micro-convex, front-side view, automatic 3D transducer. The patient was examined in dorsal lithotomy, with the hips flexed and abducted. The probe was placed on the perineum slightly anterior to the anus. Initial two-dimensional (2D) US made possible adequate definition of the region of interest between the anal margin and the lower third of the rectum. Then, 3D automatic acquisition was performed. Analysis was conducted off-line from stored 3D data, using multiplanar reconstructions, tomographic US imaging, and various volume rendering modes, including minimum intensity projection, maximum intensity projection, and static volume contrast imaging [9, 10]:

- Multiplanar reconstruction provided images of the region of interest in three perpendicular planes (axial, sagittal, and coronal; Figs. 1a, 2a, c, 3). All three planes are displayed simultaneously and can be moved and rotated to allow the operator to visualize a lesion/area at different angles and to measure them precisely in any desired direction. The unique feature of multiplanar reconstruction presentation is the display of US data similar to anatomical sections or computed tomography (CT)/magnetic resonance imaging (MRI) slices, which enables precise evaluation of the pathology in regard to anatomical structures.

Analysis of 3D data was performed independently by 2 radiologists experienced in proctological ultrasound and blinded to both each other’s evaluation and to the patient’s clinical history. Assessment of inter-observer variability showed an overall very good agreement (0.99, Kappa statistic 0.951) [29].

## Results

Four patients with high-grade anal canal stenosis were evaluated using both proctography and 3D transperineal ultrasound with a micro-convex transducer. Patient’s characteristics, results of proctography, 3D ultrasound, and treatment adopted are shown in Table 1.

**Table 1:** Proctographic and ultrasonographic findings in patients with anal stenosis

Patient No.	Grade of stenosis	Etiology	Proctography	3D Transperineal ultrasonography	Surgical treatment
1.	3rd	Radiotherapy for prostate cancer	Anal stenosis 1.5 cm long and dilated rectal ampulla	Stenosis of the distal 2/3 of the anal canal and dilated rectal ampulla. Fibrotic irregularities of the internal sphincter. Regular external sphincter	Excision of the posterior scar of the anal canal and posterior internal sphincterotomy
2.	3rd	Sphincter repair	Anal stenosis 1 cm long and dilated rectal ampulla	Anal stenosis 1cm long below the dentate line. Posterior scar involving the internal and external sphincters and adherent to the anoderm	Removal of the posterior scar and anodermal flap insertion
3.	3rd	Chronic anal fissure	Anal stenosis 3 cm long	Anal stenosis 3.5 cm long. Hypertrophic (1 cm) internal sphincter with fibrotic changes	Partial lateral internal sphincterotomy and fissurectomy
4.	2nd	Hemorrhoidectomy	Anal stenosis 3.5 cm long	Anal stenosis 3 cm long. Thickened (1 cm) internal sphincter with posterior scar involving the mucosa	Excision of the posterior scar and anodermal flap insertion

### Case 1

A 71-year-old man had a third-degree stenosis of the anal canal 3 years after radiotherapy for prostate cancer. Uropolinumproctography showed a 1.5-cm-long stenosis of the anal canal and a dilated rectal ampulla. 3D transperineal US demonstrated a stenosis of the posterior distal 2/3 of the anal canal with dilatation of the rectal ampulla proximal to the stenosis. Multiplanar reconstruction clearly showed slight fibrotic irregularities of the internal anal sphincter (Fig. 1a). Visualization of the rectum with maximum intensity projection was similar to proctography. At surgery, the posterior scar was excised and the internal sphincterotomy was performed in the same position. At 2-month follow-up, the patient had no stenosis and did not complain of fecal incontinence.

In this case, proctography and 3D transperineal US (Table 1) were concordant, showing that the whole rectum was distended and not affected by inflammation or stenosis, as may occur following radiotherapy. In addition, US demonstrated normal morphology of the external anal sphincter and only slight fibrotic changes in the internal anal sphincter. These findings were crucial for surgical planning and to avoid a colostomy, which would have been indicted in case of compromised anal sphincters.

### Case 2

A 40-year-old man had a third-degree stenosis of the anal canal secondary to reconstruction of muscles damaged by sexual assault. Uropolinumproctography showed a short (1 cm) stenosis of the anal canal and dilated rectal ampulla. 3D transperineal US revealed a 1-cm stenosis below the dentate line, and an extensive (0.6 cm^3^), posterior, hypoechoic scar, involving both the internal and external anal sphincter and adherent to the anoderm. However, no residual of or recurrent damage to the sphincters was found. Dilatation of the proximal anal canal on coronal image at multiplanar reconstruction confirmed a short stenosis. Minimum intensity projection provided images of the hypoechoic scar from different angulations (Fig. 2). At surgery, the stenosing scar was removed and an anodermal flap was inserted. At 2-month follow-up, the patient had no stenosis and did not complain of fecal incontinence.

In this case, both proctography and 3D transperineal US (Table 1) assessed the level and length of the stenosis. Additionally, 3D ultrasonography provided a preoperative evaluation of the anal sphincters, which was important in the surgical strategy of removing the scar and fashioning a flap, rather than reconstructing the sphincter muscles.

### Case 3

This patient was a 73-year-old man with a third-degree stenosis of the anal canal secondary to a chronic anal fissure. For several years, he had difficulty defecating with more recent development of significant pain. Uropolinumproctography showed a 3-cm stenotic anal canal. 3D transperineal US revealed a 3.5-cm stenosis of the anal canal with a hypertrophic internal anal sphincter (1 cm thickness) presenting an hyperechoic pattern due to fibrosis. The mucosa appeared hypoechoic; however, it did not show the folded pattern seen on normal transperineal scans (Fig. 3). Severe stenosis of the anal canal was confirmed at surgery. After dilatation, hypertrophy and fibrosis of the internal anal sphincter and a posterior chronic anal fissure were visualized. Partial lateral internal sphincterotomy with fissurectomy was performed. Recovery was uneventful.

In this case, both proctography and 3D ultrasonography (Table 1) determined the level and length of the stenosis. Additionally, 3D transperineal US provided relevant preoperative information, demonstrating abnormal inflammatory changes in the internal anal sphincter, but excluding an invasive anal cancer.

### Case 4

A 39-year-old man with a history of hemorrhoidectomy, complained of obstructed and painful defecation. On proctological examination, he had second-degree stenosis of the anal canal due to scarring, as well as a posterior fissure. Uropolinumproctography visualized a stenotic, 3.5-cm-long anal canal. On 3D transperineal US, the length of the anal canal was 3 cm and the internal anal sphincter appeared thickened (1 cm) with a posterior hyperechoic scar involving the mucosa. At surgery, anal fissure and stenosing scar were excised with the insertion of an anodermal flap. Recovery was uneventful.

In this case, proctography and 3D transperineal US (Table 1) were concordant on the level and length of stenosis. However, the ultrasonographic finding of a thickened internal anal sphincter with posterior scar guided the surgical decision to construct a flap.

## Discussion

The most relevant information needed for planning optimal surgical treatment for anal canal stenosis are the length and level of the stricture, and whether the stenosis is limited to the anus or involves the rectum as well the status of the anal sphincters. Assessment of severe stenosis with digital rectal examination or using conventional endoanal US is often unfeasible or very difficult to perform even under local or general anesthesia. Proctography may be used to measure the length of the stenosis, but it requires radiation and complex instrumentation. External phased-array MRI [30] could be a technique of choice in these patients; however, it is limited by costs and availability. Conventional 2D transperineal US has been found to be a feasible alternative to endoanal US, providing information on internal and/or external sphincter defects [28]. Our preliminary study showed that transperineal US is also a feasible, reliable, noninvasive, easy to perform, and rapid modality for the evaluation of anorectal stricture. Compared to proctography, transperineal US not only allows the measurement of the level and length of the stenosis, but also provides additional information on the integrity of the anal sphincters, the status of mucosal layer, and the presence of invasive anal cancer. The introduction of 3D US, constructed from the synthesis of a large number of 2D images, has extended the range of indications and improved the diagnostic accuracy due to multiplanar reconstruction and tomographic US imaging [10, 11, 16, 17]. In our study, the visualization of the anal canal in the axial, sagittal, and coronal planes made it possible to differentiate and measure a “low” stenosis (from the anal verge to 0.5 cm below the dentate line) due to scarring (patients 1 and 2) from a “complete” stenosis due to hypertrophy or fibrosis of the internal anal sphincter caused by fissure (patient 3) and hemorrhoidectomy (patient 4). The ultrasonographic data were fundamental for surgical planning: sphincterotomy (patients 1 and 3) and various techniques of anoplasty (patients 2 and 4), respectively.

A disadvantage of 3D transperineal US, however, is that it cannot visualize the whole rectum due to its limited field of view. For this reason, in patients with ultrasonographic evidence of a complete anal stenosis which may occur after radiotherapy or in Crohn’s disease, proctography is also necessary.

In conclusion, the results of our assessment of anal stenosis with 3D transperineal US are promising. In cases where standard digital rectal examination and/or endoanal US are unfeasible, painful or potentially dangerous for the anal sphincters, the transperineal ultrasonographic approach should be considered the technique of choice [24]. Further studies with larger series of patients are needed to confirm our preliminary findings.
